# Assessment of DHA on reducing early preterm birth: the ADORE randomized controlled trial protocol

**DOI:** 10.1186/s12884-017-1244-5

**Published:** 2017-02-13

**Authors:** Susan E. Carlson, Byron J. Gajewski, Christina J. Valentine, Lynette K. Rogers, Carl P. Weiner, Emily A. DeFranco, Catalin S. Buhimschi

**Affiliations:** 1 Department of Dietetics and Nutrition, MS 4013, University of Kansas Medical Center, 3901 Rainbow Blvd., Kansas City, KS 66160 USA; 2 Department of Biostatistics, MS 1026, University of Kansas Medical Center, 3901 Rainbow Blvd., Kansas City, KS 66160 USA; 3 Department of Obstetrics and Gynecology, University of Cincinnati, 231 Albert Sabin Way, PO Box 670526, Cincinnati, OH 45267 USA; 4 The Research Institute at Nationwide Children’s Hospital, Center for Perinatal Research, 700 Children’s Drive, Columbus, OH 43205 USA; 5 Department of Obstetrics and Gynecology, MS 2028, University of Kansas Medical Center, 3901 Rainbow Blvd., Kansas City, KS 66160 USA; 6 Division Maternal Fetal Medicine, Center for the Developmental Origins of Adult Health and Disease, 3901 Rainbow Blvd., Kansas City, KS 66160 USA; 7 Department of Obstetrics and Gynecology, Ohio State University, 370 W. 13th Ave., Rm 588, Columbus, OH 43210 USA

**Keywords:** Docosahexaenoic acid, Preterm birth, Pregnancy

## Abstract

**Background:**

Preterm birth contributes to 0.5 million deliveries in the United States (one of eight pregnancies) and poses a huge burden on public health with costs in the billions. Of particular concern is that the rate of earliest preterm birth (<34 weeks) (ePTB), which has decreased little since 1990 and has the greatest impact on the overall infant mortality, resulting in the greatest cost to society. Docosahexaenoic acid (DHA) supplementation provides a potential high yield, low risk strategy to reduce early preterm delivery in the US by up to 75%. We propose a Phase III Clinical Trial (randomized to low or high dose DHA, double-blinded) to examine the efficacy and safety of high dose DHA supplementation to reduce ePTB. We also plan for a secondary pregnancy efficacy analysis to determine if there is a subset of pregnancies most likely to benefit from DHA supplementation.

**Methods:**

Between 900 and 1200 pregnant women who are ≥ 18 years old and between 12 and 20 weeks gestation will be recruited from three trial experienced academic medical institutions. Participants will be randomly assigned to two daily capsules of algal oil (totaling 800 mg DHA) or soybean and corn oil (0 mg DHA). Both groups will receive a commercially available prenatal supplement containing 200 mg DHA. Therefore, the experimental group will receive 1000 mg DHA/d and the control group 200 mg DHA/d. We will then employ a novel Bayesian response adaptive randomization design that assigns more subjects to the “winning” group and potentially allows for substantially smaller sample size while providing a stronger conclusion regarding the most effective group. The study has an overall Type I error rate of 5% and a power of 90%. Participants are followed throughout pregnancy and delivery for safety and delivery outcomes.

**Discussion:**

We hypothesize that DHA will decrease the frequency of ePTB <34 weeks. Reducing ePTB is clinically important as these earliest preterm deliveries carry the highest risk of neonatal morbidity, as well as contribute significant stress for families and post a large societal burden.

**Trial registration:**

This trial was registered with ClinicalTrials.gov (identifier: NCT02626299) on December 8, 2015. Additional summary details may be found in Table 1.

## Background

### DHA intake and status of US women during pregnancy and physiological importance

Docosahexaenoic acid (DHA) is a long-chain polyunsaturated fatty acid member of the n-3 (or omega-3) fatty acid family. DHA is found in animal foods with the richest sources being varieties of ocean fish [1]. On average, US women consume ~60 mg DHA/day [2] but synthesize little DHA from the α–linolenic acid (18:3n-3) they consume in other foods [3, 4]. DHA intake among US women is lower than other Western populations [2]. Two commonly used indicators of DHA status: 1) red blood cell phospholipid (RBC-PL) DHA as a percent of total membrane fatty acids [5, 6] and 2) human milk DHA as a percent of total fatty acids [7] are lower in US women than in other developed countries. For example, baseline RBC-PL-DHA means ranged from 4.3 to 5.0% in our last three Kansas City pregnant cohorts [5, 8, 9] compared to greater than 6% RBC-PL-DHA reported by others [10–12]. The US rate of preterm birth (PTB) (<37 weeks) is also higher than other developed countries [13]. The NICHD Maternal Fetal Network multi-center trial notes the highest rate of PTB for women in the lowest quartile for RBC-PL-DHA (OR 1.45) [14]. Worldwide, 24% of all PTBs occur in India, where vegetable-based diets low in DHA are common [13].

**Fig. 1:**
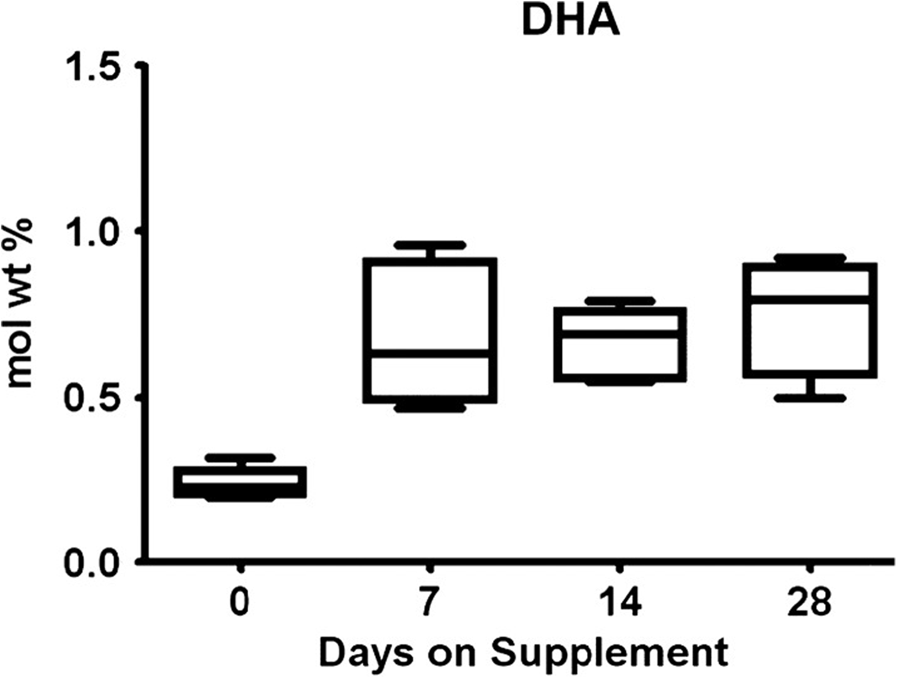
Effect of DHA supplementation on milk DHA content

**Fig. 2:**
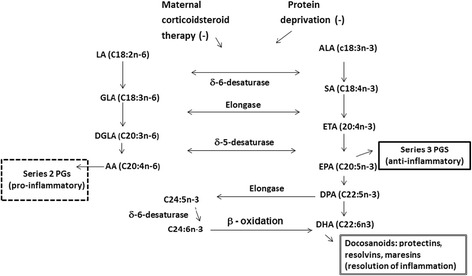
Biochemical pathways for DHA synthesis

US studies typically report a mean of 0.15–0.2% DHA in human milk [7, 15, 16] in contrast to countries and groups where ocean fish are routinely consumed (0.5 to 2.7%) [17, 18]. Our pilot feasibility trial found extremely low milk DHA in women consuming a placebo, but milk DHA increased to 0.5–0.7% in the group assigned to receive a dietary supplement of 1000 mg of DHA/day [16] (Fig. 1). Pregnant women consuming more DHA also provide more DHA to their fetus and after delivery, have higher milk DHA during lactation. It is well established that the biosynthesis of DHA from α-linolenic acid is very limited [3, 4], especially under several conditions including caloric deprivation, protein inadequacy, and corticosteroids, which inhibit the δ6-desaturase and, therefore, DHA synthesis [19] (Fig. 2). In fact, there have been many attempts to increase DHA status and milk DHA by feeding α–linolenic acid without success.

Preformed dietary DHA found in marine life, algal sources, or eggs can produce immediate biologic effects. Dietary DHA also increases DHA in membranes of cells from all organs that have been studied. Higher DHA status is linked to a number of positive health outcomes including protection against cardiovascular disease [20, 21], breast cancer [22–24], and Alzheimer’s disease [25–28] and more recently to resolution of inflammation [29–31] and neuroprotection [32, 33]. Higher DHA status during development is linked to higher cognitive performance [34–37], lower allergy [38–41], and lower adiposity [42]. In summary, DHA is found in some foods but little is synthesized from α-linolenic acid. DHA intake and status of US women are among the lowest in the world.

### Effects of DHA and EPA supplementation on gestation duration, preterm birth, ePTB and VLBW

DHA status in pregnancy was first linked to longer gestation, higher birth weight, and less PTB by early studies of Olsen and collaborators [43, 44] after they observed longer gestation among the Faroe Islanders who consumed higher DHA and EPA compared to the Danes [43]. Three recent systematic reviews address the question of pregnancy outcomes in randomized trials of total omega-3 long chain polyunsaturated fatty acid (LCPUFA) supplementation [45–47]. A 2006 Cochrane review that included six trials with 2755 women [45] found a 2.6-day increase in gestation duration favoring supplementation (*P* = 0.0009). Only one review found a significant reduction in overall PTB (<37 weeks) (*P* values = 0.03–0.29) [45–47], but all reported a reduction in early preterm birth (ePTB) (<34 weeks) with odds ratios favoring supplementation of 0.42 to 0.74. However, the ePTB findings are based on only three studies that provided DHA in amounts of 800–2000 mg/d. While two trials conducted in Australia and Europe found a reduction in ePTB [48, 49], the US trial conducted by the NICHD Maternal Fetal Network trial observed no decrease in ePTB despite supplementation with 800 mg DHA/d. The absence of an effect in this trial may be linked to the fact the trial included only women with a prior preterm birth and all received weekly injections of progesterone [50]. In contrast, only 4.9% of women in our Kansas City KUDOS study had a prior PTB and only 2.3% (*n* = 7) a prior ePTB (one had a repeat ePTB in our study). None received progesterone for short cervix. The potential of DHA to reduce ePTB is plausibly linked to its role in reducing inflammation, a final pathway for spontaneous ePTB. After the first two systematic reviews were published, our US trial was published, reporting significantly less ePTB (4.8 vs 0.6%, *P* = 0.025), fewer very low birth weight (VLBW) births (3.4 vs 0%, *P* = 0.026) and fewer days in hospital for infants born preterm (40.8 vs 8.9 day, *P* = 0.026) in the group supplemented with 600 mg/d DHA compared to placebo [5]. We conclude from studies conducted around the world that a high dose of DHA supplementation can reduce ePTB. No study has evaluated high dose DHA supplementation as a primary outcome to reduce ePTB, which we propose to do in a US population known to have poor DHA status before and during pregnancy.

### Importance of reducing ePTB and what the evidence suggests may be happening with high dose DHA supplementation

Overall ePTB rate in the US for 2012 was 3.4% [51]; however, the ePTB rate for non-Hispanic blacks (7.0%) was higher than for non-Hispanic whites (3.3%). ePTB compared to late PTB (≥34 weeks–37 weeks) carries a much greater risk of infant morbidity and mortality and costs society billions of dollars. Three systematic reviews [45–47], one conducted after publication of our trial [5] find a significant reduction in ePTB with high dose DHA supplementation. Biomarkers assessed prior to 20 week gestation suggest that the risk of spontaneous preterm labor has an early etiology from stressors linked to inflammation particularly PTB due to prolonged rupture of membranes and chorioamnionitis [52–55]. We hypothesize that DHA will prolong gestation duration for a period of time that will be sufficient for the fetus to require less medical intervention; i.e., that DHA will both reduce ePTB and improve neonatal outcome.

### Mechanisms by which DHA might reduce ePTB

DHA unlike other omega-3 fatty acids (α–linolenic acid and eicosapentaenoic acid (EPA)) uniquely modulates cell surface ligands to attenuate inflammation, demonstrated in both humans and animal models [56–60]. While the mechanism of preterm parturition remains elusive and complex [61], DHA has plausible cellular effects that could modify the onset or change the timing of inflammation [62, 63] and promote the activation of SIRT1 expression directly impacting endothelial relaxation [64], or altering membrane fluidity [64, 65] and cell signaling [66, 67]. It is now recognized that DHA released from phospholipids in cell membranes serves as a precursor for docosanoids (22 carbon mediators such as resolvins, protectins, and maresins) that are anti-inflammatory, resolve inflammation, and protect against inflammation [31, 68–72] (Fig. 2). While it is generally believed that fish oil (a source of both DHA and EPA) increases gestation duration because EPA competes with arachidonic acid (ARA), the source of the 2-series prostaglandins E_2_ and F_2α_ required for labor and delivery [73, 74], we provided only DHA in our previous trial. Moreover, the effect of DHA supplementation in our trial and the Australian trial was significant for early births only; i.e., we find no evidence DHA increases gestation in general or in any other select group of pregnancies.

A recent pilot study observed a lower preterm, premature rupture of the membranes (PPROM) in frequent fish eaters randomly supplemented with only 100 mg DHA/d [75] further linking DHA intake to reduced inflammation. We speculate that pregnancies at risk for ePTB include a disproportionate number with inflammation. sRAGE is positively linked to chorioamnionitis and preterm labor [58] and LPS-induced inflammation in a murine model [76]. In the latter example, the increase in sRAGE was attenuated by DHA supplementation. sRAGE is negatively associated with IL-6, sepsis and FIO2 requirements in PT cohorts [53, 58, 77], however, sRAGE has not been compared in term and preterm pregnancy. We propose to determine if the higher DHA supplementation influences either maternal or cord blood sRAGE as a possible mechanism of DHA PTB preventive activity.

### Rationale for DHA doses

**Fig. 3:**
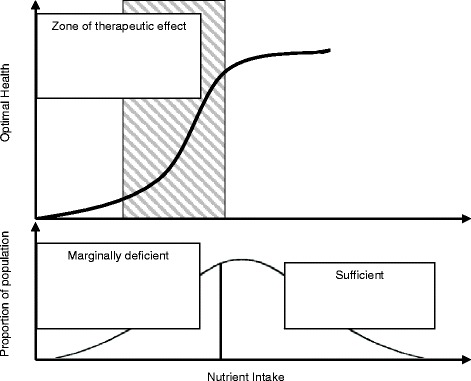
Role of nutrient status on physiological response

**Fig. 4:**
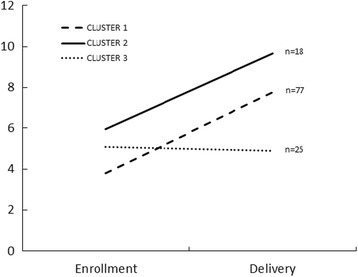
Clusters in DHA status at enrollment and birth from an earlier trial of DHA supplementation during pregnancy

The Institute of Medicine does not set a Dietary Reference Intake (DRI) for DHA in pregnancy; however, the FAO/WHO and expert groups suggest an intake of 200 mg of DHA per day for pregnancy and lactation [78–80]. Many US prenatal vitamins now contain 200 mg of DHA, and 15% of women in our trial took a DHA supplement. We propose to compare this low, recommended dose of DHA (control group) to a high dose of DHA supplement (experimental group). We chose 1000 mg as a high dose because doses up to 2000 mg/d of DHA have reduced ePTB in populations that consume more DHA than in the US [47–49]. More DHA may be helpful in the US where intake and status at baseline are low. Trials providing less than 600 mg DHA have not found a reduction in ePTB [81, 82]. DHA is a nutrient and intake and status are inherently variable. Women with low DHA status may require more DHA to reach an intake to reduce ePTB. All nutrients have an intake that is optimal for a given outcome and the ability to improve that outcome with supplementation is based on an individual’s status at baseline (Fig. 3). Overall, subjects in our previous trial had a very low DHA status (mean RBC-PL-DHA = 4.3%) consistent with DHA deficiency [83]. We identified three clusters in the DHA supplemented group of KUDOS (Fig. 4). Cluster 1 includes the majority of the DHA group and was exceedingly deficient with a mean baseline RBC-PL-DHA = 3.5%.

### Nutritional approach to reduce ePTB and VLBW

There is currently no accepted method for predicting pregnancies that will end in spontaneous ePTB and no known treatment to prevent the occurrence of spontaneous ePTB with the possible exception of progesterone therapy for women with cervical lengths of ≤20 mm [84, 85]. In contrast, to our US trial, the larger DOMInO trial conducted in Australia excluded pregnant women with serious maternal illness and found a significant reduction in ePTB with 600 mg/d DHA (*n* = 301, 0.6% vs. 4.8% of births) and 800 mg/d DHA (*n* = 2399, 1.1% vs 2.2% of births), respectively. As mentioned above, the presumed effect of DHA is reduction/resolution of inflammation, a joint mechanism for both cervical ripening and spontaneous ePTB. Even in the NICHD trial, women with the lowest DHA blood concentrations were more likely to have a recurrent spontaneous PTB [86]. In both our trial and DOMInO, the incidence of ePTB in the placebo group was similar to the incidence within the country. These trials and two systematic reviews suggest that a high dose supplement of DHA could effectively reduce ePTB. We feel such an approach could be particularly useful in pregnant US women whose DHA status is among the lowest of any population in the world. It is well known that 500–1000 mg DHA/d in adults improves clinical biomarkers of inflammation, systemic vascular changes, and endothelial cell function while low DHA intake shifts immune homeostasis towards a more pro-inflammatory response. We propose to compare 1000 mg DHA to 200 mg DHA/d. The lower amount of DHA is found in many currently available prenatal supplements, while the studies that have found an effect of DHA on ePTB have provided between 600 and 900 mg/d [5, 48, 49].

### Research objectives

The primary purpose of this multicenter, randomized, double-blind, controlled Phase III clinical trial is to determine if DHA supplements totaling 1000 mg/d compared to 200 mg/d during the last two trimesters of pregnancy can reduce spontaneous ePTB (<34 weeks gestation) (Specific Aim 1) and to conduct a secondary pregnancy efficacy analysis to determine if there is a subset of pregnancies most likely to benefit from DHA supplementation (Specific Aim 2). To obtain information about the effects of DHA on inflammation, we will measure sRAGE, which is plausibly influenced by DHA supplementation based on a murine model of LPS-induced inflammation and bank plasma samples to determine the impact of DHA on the transcriptome which broadly reflects a range of physiologic processes (Specific Aim 3). We will perform a prospective randomized comparative effectiveness adaptive design study with pregnant women to enhance efficiency of the trial and expedite results to the research community and to guide clinical practice. First analysis will be by intent-to-treat. Safety data will be collected as required for Phase III RCTs (Specific Aim 4). We will bank umbilical cord blood at delivery to be available for future evaluations. A trial summary and registration details can be found in Table 1.

**Table 1:** Trial registration data

Data category	Information
Primary registration and trial identification number	ClinicalTrials.gov NCT02626299
Date of registration	December 8, 2015
Secondary identifying numbers	R01 HD083292; IND 129482; IRB STUDY00003455
Source of monetary or material support	National Institute of Child Health and Human Development
Primary sponsor	University of Kansas Medical Center
Secondary sponsor/Collaborators	University of Cincinnati
	Ohio State University
	Nationwide Children’s Hospital
Contact for public queries	Beth Kerling, MS RD (ekerling@kumc.edu)
Contact for scientific queries	Susan Carlson, PhD (scarlson@kumc.edu)
Public title	Assessment of DHA On Reducing Early preterm Birth (ADORE) Trial
Scientific title	Docosahexaenoic Acid Supplementation in Pregnancy to Reduce Early Preterm Birth
Countries of recruitment	United States
Health conditions or problem studied	Preterm birth
Interventions	Active treatment: 1,000 mg DHA per day
	Standard of care: 200 mg DHA per day
Key inclusion and exclusion criteria	Ages eligible for study: ≥ 18 years
	Genders eligible for study: female
	Accepts healthy volunteers: yes
	Inclusion criteria: pregnant female ≥ 18 years; 12–20 weeks of gestation, agree to consume study capsules; available by telephone
	Exclusion criteria: multiple gestation, unwilling to discontinue use of another prenatal supplement with DHA, allergy to any component of DHA product (including algae), soybean oil or corn oil
Study type	Interventional
	Allocation: randomized
	Endpoint classification: safety/efficacy study
	Intervention Model: parallel assignment
	Masking: double blind
	Primary purpose: prevention
	Phase III
Date of first enrollment	June 8, 2016
Recruitment status	Active recruitment
Primary outcome	Occurrence of early preterm birth
Key secondary outcomes	Efficacy analysis for subset populations
	Effect of DHA on inflammation
	Safety evaluation

## Methods/Design

The Standard Protocol Items: Recommendations for Interventional Trials (SPIRIT) guidelines [87] are followed for design and conduct of this trial.

### Participants and eligibility criteria

Women who are 18 years and older and are in their 12^th^ to 20th week of gestation based on ACOG guidelines [88] will be eligible for enrollment. Pregnant women who receive prenatal care in obstetrics clinics of all 3 participating centers (University of Kansas Medical Center University of Cincinnati, and The Ohio State University) receive an ultrasound assessment at approximately 12 weeks gestation. The estimated date of delivery (EDD) determined by ACOG guidelines at this assessment will be fixed as the EDD for assessment of gestation duration in the study. Women must be able to read or orally understand the study in English or Spanish. They must agree at enrollment to consume the capsules assigned to them from enrollment through delivery.

Women with a multiple gestation will be excluded, as the background rate of PTB and low birthweight are higher likely for reasons other than hypothesized with DHA. The availability of a telephone is necessary for optimal coordination of both phases of the study. We routinely obtain additional phone numbers of friends and family who have a stable address. The complete inclusion and exclusion criteria are illustrated in Table 2.

**Table 2:** Inclusion and exclusion criteria

Inclusion criteria	
1.	Pregnant females 18.0 years and older 12 to 20 weeks of gestation at study entry
2.	Agree to consume study capsules and a typical prenatal supplement of 200 mg DHA
3.	Available by telephone
4.	English or Spanish speaking
Exclusion criteria	
1.	Less than 18 years of age
2.	Expecting multiple infants
3.	Gestational age at baseline <12 weeks or >20 weeks
4.	Unable or unwilling to agree to consume capsules until delivery
5.	Unwilling to discontinue use of another prenatal supplement with DHA
6.	Women with allergy to any component of DHA product (including algae), soybean oil or corn oil

### Recruitment and enrollment

Potential participants will be approached by trial recruiters in the ambulatory units of each study site if a preliminary evaluation suggests they meet the inclusion and exclusion criteria. The goal is approximately equal enrollment at the 3 centers with up to 400 subjects enrolled per center (depending upon the dictates of the Bayesian response adaptive randomization). We expect to enroll 2–3 subjects per week at each of the three centers (6–9 subjects/week total) and anticipate 11% will be lost over the course of the study.

Subjects who consent will be asked to provide blood on the day they are enrolled for fatty acid analysis of RBC-PL fatty acids, sRAGE and to bank plasma, serum and white cells for future nutrient and marker analysis. A urine sample will be collected for measurement of endocrine disrupting chemicals. Participants will complete the National Cancer Institute Diet History Questionnaire (NCI DHQ-II) to obtain information regarding maternal dietary intake, and additional questions will be asked regarding nutritional supplement intake prior to and during pregnancy. Medical and social histories will also be obtained and tracked throughout pregnancy and delivery. A complete schedule of participant events is illustrated in Table 3.

**Table 3:** Participant schedule of events

	Enrollment	Treatment				Delivery	Postpartum F/up
	Baseline Visit	Enroll F/up	Refill Contacts	Mid-Pregnancy	Pre-delivery	Hospital Visit	Delivery F/up
Timeline	12 to <20 weeks GA	Up to14 days after enrollment	~ every 6 weeks after supplement dispensed	28–36 weeks GA	PRN as EDD nears	During hospital admission	30 days after delivery^a^
Informed Consent	✓						
Verify Inclusion/Exclusion criteria	✓						
Health History	✓						✓
Dietary Supplement Intake	✓						✓
DHA FFQ	✓						
DHQ-II FFQ	✓						
Measure Maternal Height	✓						
Maternal Blood Draw	✓					✓	
Maternal Urine Sample	✓			✓ 33–36 weeks GA			
Randomization and dispense supplement bottles	✓	Start supplements day after enrollment Continue daily intake until delivery					
Review of Maternal Medical Records	✓			✓		✓	
Telephone Contact		✓	✓		✓	✓	✓
Mail Investigational Supplement			✓				
Return Investigational Supplement			✓				✓^b^
Delivery Information (if needed, Blood Kits)				✓			
Review of Infant Medical Records						✓	
Cord Blood Collection						✓	
ROI forms	✓					✓	
Participant Compensation	✓					✓	
Adverse Events	✓	Record as they occur after enrollment					

^a^Delivery Follow-up Contact will be made 30 days after delivery or after mom and baby have been discharged from the hospital if later than 30 days

^b^Investigational Pharmacy will mail participant envelope to return all study capsules after delivery

### Randomization and implementation

A maximum number of pregnant women n_max_ = 1200 (we plan for 1355 enrollments due to expected dropout) will be randomized to one of two arms, either algal oil as a source of DHA (800 mg DHA in two capsules) or placebo oil (two capsules of half soybean oil and half corn oil, without DHA). In addition, all participants will receive a prenatal supplement with 200 mg DHA and required not to use another prenatal with DHA.

Each study site location will have a separate randomization code. Using a Bayesian Adaptive Design, a decision will be made at each interim analysis. The randomization structure will be updated depending upon the birth outcomes. The primary endpoint, the percentage of spontaneous ePTB, will drive the adaptive randomization. The data will be analyzed after 150 particpants are enrolled in each group and the randomization schedule updated. The arm that looks to be the best will get more pregnant women allocated to it in this subsequent randomization. A new adaptive randomization schedule will be generated every 13 weeks using up to date outcome data until the trial is stopped. Both the initial and subsequent updated tables will be attached to our eResearch tool [89]. Steps for randomization in eResearch include:

- ❖ Subject demographics are entered
- ❖ Subject is attached to study (at this point patient status could be screening or pre-screening)
- ❖ Verification of inclusion and exclusion criteria and documentation of the informed consent are entered into the patient randomization form triggering automatic assignment of a patient study ID and randomization to the next particular arm from the allocation table

We will test if the randomization is effective by ensuring the groups are similar at baseline (pre-randomization variables) for all such predictor variable and will be presented in a table per CONSORT guidelines [90].

### Placebo and DHA supplementation

A marine algae oil source of DHA (DSM, Columbia, MD) will be provided in capsules. Specific capsules to be used are equivalent to Spring Valley Algal-900 DHA Dietary Supplement Softgels, 450/mg per capsule. The algal oil capsules in this study provides 800 mg DHA in two (2) 1-g capsules and the higher DHA group of subjects will be asked to consume two capsules per day. The placebo control group will receive two (2) 1-g capsules containing half soybean oil and half corn oil. The soybean and corn oil combination does not contain DHA. Two capsules provide 80 mg of α-linolenic acid, a precursor of DHA. On average, US adults consume ~1000 mg/d of α-linolenic acid but can make only about ~40 mg DHA/day. Both capsules will be prepared and provided with orange flavor to mask the taste if there is eructation. The placebo and masked DHA capsules will be provided in bottles of 100 capsules (a supply for 50 days). DSM (Columbia, MD) will donate the capsules and the 200 mg DHA capsules to both groups for daily use. The latter are available commercially as a prenatal supplement marketed under several product names. The 200 mg capsules will be provided in bottles of 135 capsules (135-day supply) and will be marked with an expiration date. Other fatty acids found in the capsules do not contribute significantly to the amounts in the diets of US women. DHA is the only fatty acid expected to change in the RBC-phospholipids (PL) of the supplemented group. Patient compliance will be monitored and encouraged; in our previous trial, on average 74% of capsules were consumed.

### Capsule records and accountability

The Investigational Pharmacy at The University of Cincinnati will mail capsules to each enrolled participant on a regular schedule until delivery when the bottles and any remaining capsules are to be returned by mail to the Investigational Pharmacy in a self-addressed envelope provided with capsule delivery. The remaining capsules will be counted, the number recorded and the capsules destroyed. Records of capsules mailed to and received back from subjects will be entered into the study database with a flag to investigators at the subject’s study site. Investigators at each site will review the database and contact participants who do not return their capsule bottle. Study personnel will contact the participant by telephone early within the first month and monthly thereafter to determine if there are any problems and encourage compliance. The study investigator, study site staff and participants will not know which study arm capsules are being consumed by any patient. The Investigational Pharmacy at the University of Cincinnati will receive all bottles of capsules directly from DSM and will maintain packing receipts for study products.

### Blood collection

Maternal blood samples will be collected at enrollment and the morning following delivery by venipuncture. At enrollment and delivery, two 4-ml potassium–EDTA tubes will be obtained (BD Vacutainer, Franklin Lakes, NJ), placed on ice immediately and processed within 24 hours. Plasma, buffy coat and anticoagulated RBCs will be separated by centrifugation (3000×g, 10 min, 4 °C). At the University of Kansas Medical Center, an additional one 7-ml potassium-EDTA tube and one 5-ml serum tube will be collected at enrollment and delivery for the cell free plasma RNA analysis. Two (2) 4-ml potassium-EDTA tube of umbilical cord blood will be obtained at delivery and processed similar to maternal samples. All samples will be stored snap frozen in nitrogen and stored at −80 °C for the planned or future studies.

### Fatty acid analysis

Fatty acid content in red blood cells (RBC) will be analyzed by gas chromatography. Briefly, an aliquot of packed RBCs is extracted with organic solvents and the dried under a nitrogen stream before transmethyation with boron trifluoride-methanol [5]. The fatty acid methyl esters are extracted into organic solvent, dried under a nitrogen stream and reconstituted in dicholoromethane for analysis on a gas chromatograph with flame-ionization detection equipped with an autosampler using a fused silica capillary column (SP2560, 100 m × 0.25 mm id × 0.25um film thickness). Helium is used as the carrier gas. Fatty acid analyses will be completed at the University of Kansas Medical Center. RBC-DHA and other fatty acids are reported as weight percent of total fatty acids. RBC-DHA can also be used to evaluate compliance.

### sRAGE analysis

sRAGE will be determined at Nationwide Children’s Hospital in Columbus, OH on batched samples using ELISA-based format (MesoScale Discovery, Rockville MD) according to the protocols of the manufacturer.

### Cell free plasma RNAs

One 7-mL maternal blood sample in potassium-EDTA tube (lavender top) and one 5-ml maternal blood sample in serum tube (red top) will be collected from the subject at enrollment and delivery at KUMC to first determine their a priori risk of experiencing spontaneous preterm birth by measuring a panel of cell free RNAs, and secondly to determine the impact of DHA of the cell free plasma transcriptome. The four markers used in this panel appear in in vitro studies to alter myometrial quiescence. In validation studies of maternal samples from pregnant women at gestational ages similar to the current study, the spontaneous preterm labor panel has a sensitivity of 100%, specificity of 95%, positive and negative predictive values of 95 and 100% respectively for spontaneous PTB less than 32 weeks of gestation (unpublished, CP Weiner). Should the administration of DHA alter the predicted spontaneous PTB rate in participants with an abnormal panel, it would provide insight as to the mechanisms by which DHA might work.

### Dietary and nutrient intake

The National Cancer Institute Diet History Questionnaire (DHQ-II) is the current version of a food frequency and portion questionnaire. The database associated with the DHQ-II is based on the National Health and Nutrition Examination Surveys (NHANES) data collection from 2001 to 2006. Participants will complete the DHQ-II at enrollment using electronic/tablet entry. Paper copies will be available for Spanish speaking participants and English speaking participants that reject electronic entry. The study teams at each site will provide instruction as to how to complete the questionnaire and review DHQ-II results for completeness. Additional study staff may follow up with the study participant by telephone after enrollment if there are concerns regarding missing data, inconsistences, etc.

The project is registered at the NCI website and all electronic data will be stored securely under the PI user name and password until study end. Data will be analyzed using the Diet-Calc software. The DHQ-II provides data for 176 nutrients, dietary constituents and food groups.

In addition to the DHQ-II, the DHA Food Frequency Questionnaire will be administered. This questionnaire includes targeted questions to assess the intake of omega-3 fatty acids accurately [91]. Results of the questionnaire will be entered into the CRIS database.

### Urine collection

One non-sterile urine sample (minimum collection of 8 mL) in a 4 oz. specimen collection container will be obtained between 12 and 20 weeks of gestation ideally at the time of enrollment and again during the 3^rd^ trimester. The sample will be divided into four 2.0 mL cryovials using a disposable transfer pipette. Cryovials will be labeled and frozen at −80 °C until analysis. The levels of endocrine disrupting chemicals (EDC) will be quantified and any differences in maternal urine levels between groups determined.

### Data collection and integrity

Phase III Clinical Trials have a formal system for data collection and scrutiny in accordance with Good Clinical Practices (GCP) and conform with the regulatory requirement(s) [92]. Data collection and entry for all aspects of the study will be performed by persons at each site. Study staff will adhere to trial-specific Stand Operating Procedures (SOPs) approved by trial PIs. Clinical teams at each site will maintain essential source documents including clinical and hospital reports.

The data generated by this project will be entered at the individual site into a CRF Part 11 compliant, secure data system developed and managed by Dr. Gajewski’s team in Biostatistics. The data will be fully accessible to the other PIs at all times. An electronic case report form that includes historical information obtained from subjects not in the hospital or from their clinical record (e.g., detailed smoking history, alcohol use – number and type of alcoholic beverages before and during pregnancy using the Nutrition Educators of Health Professionals tool and DHQ-II obtained at study entry) http://www.ich.org/fileadmin/Public_Web_Site/ICH_Products/Guidelines/Efficacy/E6/E6_R1_Guideline.pdf will be maintained as the primary source document for this information. Whenever possible, site staff will use a two-pass approach to confirm accuracy of social/historical information not in the hospital or clinical record (i.e., staff will review data entry with subjects in person or by phone interview). One hundred percent of data for the primary outcome and safety (mother and newborn) and a portion of other data collected will be double-checked by the clinical teams after the data are entered into the eResearch by reviewing source documentation. The proportion of secondary outcome data will be determined by the PIs based on the actual incidence of errors observed in data entry. These master files will be established for each subject and maintained for the duration of the trial and retained according to the appropriate regulations.

Accuracy, completeness, legibility of work documents and timeliness of the data reported in the patient’s electronic case report form will be assured by the PI. Source documentation supporting the case report form data will document the dates and details of study procedures, adverse events and patient status. Any discrepancies will be explained by a note to the individual file and changes or corrections to the electronic case report form dated, initialed and explained (if necessary). Original data will not be obscured.

The trial analyst will ensure data validity and accuracy by performing edit, logic and range checks on the study database and sending queries for resolution to the clinical team. Once all database queries have been resolved by the clinical teams, the trial analyst will create the data sets for analyses (interim and final). In collaboration with Director of Research Information Technology, the analyst will finalize the study binder, which will contain copies of the annotated project case report forms, the final data dictionary, and copies of the electronic data files.

**Primary Efficacy Outcomes**

- ❖ Early preterm delivery (ePTB, <34 weeks gestation) based on ACOG guidelines

**Secondary Efficacy Outcomes**

- ❖ VLBW (<1500 g) and low birth weight (<2500 g) as recorded in hospital record
- ❖ Participant DHA status (RBC-PL-DHA) at enrollment and birth; fetal DHA status at birth from cord blood
- ❖ Gestational age (days) at delivery based on EDD in clinic record recorded following ultrasound on or before ~14 weeks gestation
- ❖ Birth weight (g), length (cm) and head circumference (cm) at delivery as recorded in hospital record
- ❖ Preterm birth (<37 weeks) based on ACOG guidelines
- ❖ Extreme preterm birth (<33 weeks)
- ❖ Pregnancy outcomes: gestational diabetes, pre-eclampsia, C-section, spontaneous or induced labor, occurrence and reason for non-routine hospitalization

### Attrition

If a patient withdraws from the study prematurely, the assessments described at delivery that apply will be obtained if available and the participant has not requested her data not be obtained. This, and the requirement to obtain medical records for adverse events during pregnancy and following birth of the infant, will be explicit in the consent form. If the participant is withdrawn due to an adverse event(s), the patient will be monitored until the adverse event has resolved or until the event is determined to be due to a stable or chronic condition. The reason for patient discontinuation will be documented. If a patient withdraws from the study, the patient’s study number will not be reassigned.

### Study termination

The study will be stopped when there is a Pr > 0.995 that the groups are different or terminate when 1355 women have been randomized (n_max_ =1200 completers after expected dropout). The data safety monitoring board (DSMB) may terminate the study if in the opinion of the safety monitor there is a determination of unexpected, significant, or unacceptable risk to the patient, however, this is not anticipated given that safety concerns have not arisen in other clinical trials in pregnant women that provided large amounts of DHA. Any action taken to suspend or terminate the project by the DSMB will be reported to the central Institutional Review Board and the NIH Office of Sponsored Projects and the program director at NIH.

## Statistical issues

### Bayesian adaptive design

We chose this design with efficiency in mind. There is broad acceptance that Bayesian Adaptive Designs save time and money and lead to more ethical studies [93]. The time is right for the use of Bayesian Adaptive Designs in comparative effectiveness clinical trials. Both the Patient Centered Outcomes Research Institute (PCORI), a leader in comparative effectiveness research, and the FDA have adopted policies/guidelines encouraging their use [94]. Using adaptive randomization (being able to change how we assign patients to the drugs during the study based on information gained during the study) allows for substantially smaller sample sizes and provides better conclusions about what group is the most effective, because it allows changes to our approach or to stop the study early if strong results are found before the scheduled end of the study [93]. We conducted extensive trial simulations comparing different designs measuring the resources (time and number of patients required) and the ability to draw important conclusions about relative efficacy of the two groups and selected the proposed design as the most effective and efficient. The following sections focus on different issues and detail how we determined power, sample size, and duration of this trial.

### Summary of the Bayesian adaptive design

We will begin interim analysis once 150 subjects have been enrolled in each group. Thereafter, interim analyses will occur every 13 weeks with data used on all births (intent-to-treat). There are many parameters that go into a Bayesian Adaptive Design but highlights include after 800 subjects have been enrolled, at each interim the stop for success is if probability (group *j* is best) > 0.995 for either group and the updated allocation probabilities are based on information weighing.

### Statistical model

The statistical model will evaluate final determination of which group is “best.” This is referred to as the group having the lowest rate of ePTB. For this study the number of PTBs is modeled with a binomial distribution. For the *j* ^th^ group the number of ePTBs, *Y*_*j*_, is modeled conditional on the number of births, *n*_*j*_, as a binomial distribution *Y*_*j*_ ~ Binom*(n*_*j*_ *,θ*_*j*_ *). θ*_*j*_ is the rate of early preterm births (ePTB) of which we provide “weakly informative” priors, logit(*θ*_*j*_ *) ~ N(−3.5,1.5* ^*2*^ *)*. This prior not only provides a design Type I error rate of 5% (see below), it is also very diffuse since the point estimate of *θ*_*j*_ is 2.9% ePTB and 95% interval 0.16–36%. This is very, very spread out. Using the data and the prior probabilities, we then use Markov Chain Monte Carlo computations to obtain the Bayesian posterior distributions of *θ*_*j*_ respectively for each group as well as calculating probability (group *j* is best).

### Response adaptive randomization

After 150 subjects are enrolled in each group the next round of subjects is randomized using a formula that takes advantage of the information gained from our analyses up to that point. Next subjects are randomly allocated to be enrolled in the *j* ^th^ group proportional to *V*_*j*_ = [prob(group j is best)xVar(*θ*_*j*_)/(*n*_*j*_ + 1)]^1/4^. This formula assigns more patients to the most promising group. The study remains blinded throughout. In response to reviewer concern that adjustment after 150 enrollments in each group would be too soon, PIs explored an alternative adaptive design beginning after 300 enrollments in each group. The sample size changed from 938 with 60% in the winning group to 940 with 56% in the winning groups. The average recruitment length changed from 184 to 183 weeks. We chose to stay with adjustment after 150 enrollments in each group because it resulted in more subjects on the better dose than the later adjustment.

### Power, sample size, trial duration, and allocation

For the purposes of this investigation we looked at several virtual (or “pretend”) responses to determine the power, sample size, time (duration), and subject allocation needed for our study. We created several scenarios for ePTB rates. We performed five sets of trial simulations based on the various combinations of response. Each set involved 100 trial simulations. We highlight two scenarios here. The first uses a slightly more conservative result than the KUDOS trial to predict what we believe is the most likely response (scenario #1 in Tables 3 and 4). If there is a best group in terms of ePTB rate, we estimated (identified) that 82% of the simulated trials had early success and 8% had late success. This trial scenario had 90% power and the sample size of this trial scenario was on average 938 (60% of these in the winning group). The average length of this trial scenario was 184 weeks. While a conventional equal randomization trial would have 90% power, it would be larger (1200 subjects), slower (230 weeks), and have a lower rate of subjects on the winning group (50%). The second is the scenario that serves as our null hypothesis (scenario #5). In this scenario there is no difference in ePTB between the groups. Therefore, the extent to which this scenario is “successful” actually reflects our Type I error rate. For this scenario, we estimated (identified) that 5% of the simulated trials had early success, 0% late success. Thus, this trial scenario produced an appropriate expected Type I error (α = 5%). The sample size of this scenario on average was 1188 subjects (equally allocated across groups). The average length of the trials under this scenario was 231 weeks.

**Table 4:** Simulated trial operating characteristics

Scenario	%.	%.	Power	Mean			Mean trial
	Finish	Finish		Subjects	% Group 1	% Group 2	(Weeks)
	Early	Late					
#1. very likely (4 vs 1%)^a^	82%	8%	90%	938	40%	60%	184
#2. likely (3 vs 0.5%)	84%	7%	91%	934	41%	59%	184
#3. unlikely (3 vs 1%)	52%	11%	63%	1046	44%	56%	204
#4. very unlikely (3 vs 2%)	23%	4%	27%	1142	46%	54%	221
#5. no difference (3 vs 3%)	5%	0%	5%	1188	51%	49%	231

^a^Based on our planned enrollment and US 2012 ePTB rates of black and white pregnancies, we anticipate 4.1% ePTB in the control group

### Primary and secondary pregnancy efficacy analysis (specific Aim 1)

The pregnancy analysis has two overall phases. The first phase of analysis (primary) investigates, using Bayesian posterior probability (group *j* is best, whether there is a simple difference between groups (no adjustment of predictor variables). A similar continuous Bayesian model will test sRAGE differences between groups. The second phase of analysis (secondary) uses Bayesian multiple logistic and continuous regression for detailed investigation as to why there are differences between groups.

All analyses will be conducted under intent-to-treat principles. Subjects will remain in the group for which they are randomized regardless of compliance. The consent form will state that medical records may be obtained from the clinic and hospital (for mother and baby) unless a specific request in writing disallowing us to obtain records is received. Nevertheless, we do anticipate some missing data. To handle missing data, logistic regression will evaluate missing data patterns as a function of subject demographics. The two categories will be “drop-out” and “did not drop out.” This analysis will help us understand the missing data pattern (missing at random, missing completely at random).

### Pregnancy efficacy analysis according to intent-to-treat principles

In the first phase of analysis, we test the differences between groups for the primary efficacy outcome (ePTB) and its subgoup (sPTB). The approach is repeated for secondary outcomes using Bayesian logistic and continuous regression (VLBW (<1.5 kg), maternal RBC PL DHA) with no covariates.

### Pregnancy efficacy analysis controlled for potential predictor variables

The goal here is to find out how well DHA supplementation predicts our primary and secondary efficacy outcomes after controlling for potential predictor variables. In the second phase of analysis, using multiple linear regression (logistic and continuous), we explore the relationships among predictor variables for regression and all primary and secondary pregnancy outcomes. The predictor variables represent five general classes: overall DHA intake, diet (intake of nutrients and foods analyzed by principal component analysis), environment, subject demographics, and maternal medical history. For exploratory purposes, the most important relationship is between the DHA dose and pregnancy outcomes. Notice that instead of a grouping variable representing groups, we utilize DHA in the form of several predictor variables, depending on their source.

- ❖ A final exploratory analysis investigates the impact of capsule intake on outcome as mediated by maternal RBC-PL DHA. Using a reasonable set of predictor variables from the regressions above, we will run two sets of regressions for each outcome variable. This will allow maternal RBC DHA to be a mediator. First we regress the RBC-PL DHA level on all appropriate predictor variables (as above). Then we will run a regression of outcome variables on RBC-PL DHA level and all other appropriate predictor variables (as above but with RBC DHA added). In this way, we are running a path analytic model where we can obtain direct effects of variables and indirect effects of variables through mediator plasma level.
- ❖ We will investigate local model adequacy in all regression analyses by exploring standardized residuals and leverage points via Cook’s distance. Possible co-linearity among predictor variables will be examined with Pearson’s correlation coefficient and variance inflation factors (VIF). Scatter plots and histograms will also be used to investigate the adequacy of the model assumptions.
- ❖ Of substantive interest in the regression analysis is that race is one of the predictor variables. Since we anticipate 22.5% of the subjects to be Black American of African descent, we can test whether efficacy of pregnancy outcomes are different for Black American women of African descent relative to other races.
- ❖ For regression analysis purposes, the pregnancy outcomes are separated into two classes of variables, either continuous or dichotomous. For the continuous variables, Bayesian regression based on the normal distribution will be utilized. For the dichotomous variables, logistic regression will be utilized.
- ❖ For all outcomes, we set the DHA dose as a predictor variable and then fit all possible subsets of the other predictor variables to explore, for the particular pregnancy outcome, which model is the best. We will utilize a global fit index called Deviance Information Criteria (DIC) to determine which variables to keep in the final model. The DIC is very general and can be used for normal and logistic regression analyses.

### Potential predictor variables for regression analysis

Class 1: Overall DHA intake

- ❖ DHA dose (capsules taken multiplied by the DHA in the type of capsule consumed)
- ❖ maternal RBC-PL DHA level at enrollment and delivery (g DHA per 100 g total fatty acids) by chromatographic analysis
- ❖ umbilical cord RBC DHA by chromatographic analysis
- ❖ estimated DHA intake at enrollment from DHQ-II and frequency/amount of consumption of food and supplement sources containing DHA

Class 2: Diet

- ❖ estimated DHA intake at enrollment from DHQ-II and frequency/amount of consumption of food and supplement sources containing DHA
- ❖ intake of other nutrients or foods, e.g., macronutrient quantity or quality, micronutrient quantity at enrollment

Class 3: Exposure to environment

- ❖ tobacco exposure prior to and during pregnancy by subject report
- ❖ alcohol intake prior to and during pregnancy by subject report defined as standard drinks/day (Nutrition Educators of Health Professionals Teaching Tool)
- ❖ measurement of endocrine disrupting chemicals (EDCs) in urine at 12–20 weeks and 33–36 weeks of gestation
- ❖ potential exposure to EDCs in environmental by questionnaire at 12–20 weeks and 22–26 weeks of gestation

Class 4: Subject demographics

- ❖ marital/relationship status, by subject report
- ❖ household income by subject report
- ❖ insurance type (private, public, uninsured) by review of clinic/hospital record
- ❖ maternal and paternal education by subject report
- ❖ maternal age at enrollment (years) from DOB listed in clinic/hospital record
- ❖ maternal and paternal race/ethnicity from clinic record or subject report
- ❖ fetal sex

Class 5: Maternal medical history

- ❖ BMI calculated from the measured prenatal clinic weight record and measured height during the first prenatal visit, or if missing, the self-reported pre-pregnancy weight
- ❖ gestational weight gain (last clinic visit in pounds minus 1st measured weight or pre-pregnancy weight)
- ❖ gestational age at enrollment (days) calculated from EDD (based on ACOG guidelines)
- ❖ reproductive history
- ❖ characteristics of previous pregnancies (early preterm birth, pre-eclampsia, gestational diabetes)
- ❖blood pressure throughout pregnancy
- ❖ iron status/hemoglobin at enrollment and mid-pregnancy
- ❖ cervical length between 18 and 22 weeks of gestation if reported
- ❖ estimated blood loss at delivery by estimate of the deliverer
- ❖ infant APGAR scores
- ❖ meconium in amniotic fluid
- ❖ evidence of illicit drug use from clinic/hospital record

### Safety monitoring

A DSMB composed of two neonatologists, one pediatric pharmacologist and one pediatric epidemiologist will meet yearly and generate a report to the PIs and IRB. The medical monitor will chair DSMB discussions. They will receive all reportable adverse events (i.e., events that are unexpected and related or probably related) within five working days after the investigators learn of the event. They will also be provided a complete list of all AEs by the trial analyst prior to each DSMB meeting. The medical monitor may request the actual product assigned to an individual if needed. After all data for the study have been monitored, entered, cleaned and locked, a safety report will be generated by the study analyst with input from the medical monitor to generate the safety report for publication.

### Responsibilities and management plan

The submission of this proposal with Drs. Susan E. Carlson, Christina Valentine and Byron Gajewski as principal investigators represents a unique collaboration of three scientists with a strong interest in the nutrient DHA but representing different key disciplines and experiences to work together to conduct a clinical trial that requires several clinical sites to meet the planned recruitment goal needed to test the hypothesis that DHA can reduce early preterm birth (ePTB). Dr. Carlson and Dr. Byron Gajewski have collaborated on an NIH funded trial (R01 DHA and Pregnancy Outcome) since 2005. The two are also working in collaboration with the PIs from the DOMInO trial in Australia to determine women who most benefited from DHA supplementation to reduced ePTB in that trial; and have generated preliminary data shared in this proposal. Dr. Valentine is a neonatologist who initially trained as a dietitian who has conducted two trials in maternal dietary DHA and human milk composition that have an FDA IND. She has worked in the field of neonatology as a physician since 2002 and has a goal of improving perinatal nutrition. She is a co-investigator on an NIH funded R01 trial from the Office of Dietary Supplementation to examine the inflammatory homeostasis of preterm infants after maternal DHA supplementation. She was previously at Nationwide Children’s Hospital and The Ohio State for 9 years and maintains a strong collaboration with her previous mentor Dr. Lynette Rogers who is site PI at Nationwide Children’s Hospital.

For the current proposal, Dr. Gajewski’s role as a PI is to design and govern the Bayesian Adaptive Design. He has expertise in the design and implementation of Bayesian adaptive designs. He has published new Bayesian clinical trials methodology in a top tier biostatistics journal (Statistics in Medicine), of which one was quoted in NHLBI’s RFA-HL-08-013. He has also published two papers showcasing novel Bayesian predictors of clinical trials accrual with co-PI Carlson. He was also successful in gaining PCORI (CER-1306-02496) funding using a novel Bayesian adaptive design. His experienced team in Biostatistics will also set up and manage a common secure data entry system for the three study sites and be responsible for generating the initial and subsequent (adaptive) randomizations that will be used by the Investigational Pharmacy at the University of Cincinnati to allocate the supplement to subjects at all three sites. He will be responsible for the randomization, setting up a common secure data entry system for the three proposed study sites (University of Cincinnati, Ohio State University, and the University of Kansas Medical Center) and for managing the adaptive design. With his Co-PIs, Carlson and Valentine, he will be primarily responsible for the statistical analysis for the study.

Dr. Carlson will interface with a study coordinator at KUMC and Dr/Valentine with the study coordinators responsible for managing both the Cincinnati and Columbus, OH sites. Monthly teleconference will be used to discuss any issues of recruitment and retention at any site. The teleconference will include the PIs (Valentine, Gajewski, Carlson) and the key personnel at UC, OSU and Nationwide (DeFranco, Buhimschi, Rogers) the trial administrator (Kerling) and study coordinators at each site (Thodosoff, Smith, Caldwell). Because we have already conducted a nearly identical intervention at Kansas City, we do not anticipate a lot of new issues. We have developed a system and understand the workload engendered by the proposed study and so could be helpful in advising Dr. Valentine what will be needed for the two Ohio sites. The teleconferences will be used to review and provide updates on enrollment, testing, and data collection over the previous month provided by the leaders of each of the teams. Carlson, Valentine and Gajewski will communicate at least weekly through email or telephone conversation. Kansas City and Cincinnati are only ~90 min apart by air. The PIs will meet face-to-face 6 months into the recruitment and yearly or as needed after that. The PIs have already developed open channels of dialogue that are exercised frequently in the writing of this proposal. Bridge lines, video conferencing, e-mail and shared digital access systems are available as needed.

### Data access, dissemination, and authorship

Typically, with longitudinal projects like this one, decisions as to how and when to publish empirical reports is a difficult one. To resolve this issue, the PIs will map out a preliminary dissemination plan that is principled yet flexible enough to allow for the clearest manner of presenting the results, and to determine the extent of authorship.

## Abbreviations

ACOG: American College of Obstetrics and Gynecology
DHA: Docosahexaenoic acid
DSMB: Data safety monitoring board
eCRF: Electronic case report form
EDD: Estimated due date
ePTB: Early preterm birth
LCPUFA: Long chain polyunsaturated fatty acid
PROM: Premature rupture of membrane
PTB: Preterm birth
RBC-PL: Red blood cell phospholipid
VLBW: Very low birth weight
